# A Morphometric Assessment of the Intended Function of Cached Clovis Points

**DOI:** 10.1371/journal.pone.0030530

**Published:** 2012-02-10

**Authors:** Briggs Buchanan, J. David Kilby, Bruce B. Huckell, Michael J. O'Brien, Mark Collard

**Affiliations:** 1 Human Evolutionary Studies Program and Department of Archaeology, Simon Fraser University, Burnaby, British Columbia, Canada; 2 Department of Anthropology, University of Missouri, Columbia, Missouri, United States of America; 3 Department of Anthropology and Applied Archaeology, Eastern New Mexico University, Portales, New Mexico, United States of America; 4 Maxwell Museum of Anthropology and Department of Anthropology, University of New Mexico, Albuquerque, New Mexico, United States of America; University College London, United Kingdom

## Abstract

A number of functions have been proposed for cached Clovis points. The least complicated hypothesis is that they were intended to arm hunting weapons. It has also been argued that they were produced for use in rituals or in connection with costly signaling displays. Lastly, it has been suggested that some cached Clovis points may have been used as saws. Here we report a study in which we morphometrically compared Clovis points from caches with Clovis points recovered from kill and camp sites to test two predictions of the hypothesis that cached Clovis points were intended to arm hunting weapons: 1) cached points should be the same shape as, but generally larger than, points from kill/camp sites, and 2) cached points and points from kill/camp sites should follow the same allometric trajectory. The results of the analyses are consistent with both predictions and therefore support the hypothesis. A follow-up review of the fit between the results of the analyses and the predictions of the other hypotheses indicates that the analyses support only the hunting equipment hypothesis. We conclude from this that cached Clovis points were likely produced with the intention of using them to arm hunting weapons.

## Introduction

Caches—tightly clustered deposits of artifacts that contain little or no manufacturing and maintenance debris and that appear to have been deposited at the same time [1]—are a striking feature of the Clovis archaeological record. To date, 17 Clovis caches from the western United States have been published [1], [2]. Two assemblages from farther east—Rummells-Maske (Iowa; [3], [4]) and Lamb (New York; [5])—are often described as Clovis caches, but it is unclear whether they meet the established criteria for assignment to Clovis or whether they are in fact caches [1]. At the moment, the temporal range of Clovis caches is poorly understood. This is because only two of them—the Anzick cache (Montana) and the East Wenatchee, or Richey-Roberts, cache (Washington)—have been dated radiometrically. The usual practice is to treat caching as a part of the Clovis behavioral repertoire for the entire span of Clovis, which is widely accepted to be 13,600–13,000 calBP [6], [7].

Currently, opinions differ regarding the intended function of points found in Clovis caches. Some researchers argue that the resemblance of cached Clovis points to smaller Clovis points that have clearly been used for hunting suggests that the former were created to be used as parts of hunting weapons and were simply stored for future use [8]–[11]. Other researchers contend that points in some of the caches were produced for use in rituals rather than for hunting [12]–[17]. Additional functions for cached points have been proposed on the basis of the points included in the East Wenatchee cache. For example, Buchanan [18] and Kilby [1] have suggested that the East Wenatchee points may have been used in costly signaling displays, whereas Lyman et al. [19] have argued that the points from the East Wenatchee cache are too large to have been used for hunting and were actually designed to be used as saws.

Here we report a study in which we used a sample of points from Clovis caches and points from Clovis kill and camp sites (hereinafter “kill/camp points”) to test the hypothesis that cached Clovis points were intended to be used as parts of hunting weapons. We focused on the hunting equipment hypothesis because it is the least complicated of the hypotheses that have been put forward to explain cached Clovis points, and because it makes straightforward predictions regarding the morphological similarities and differences between cached points and kill/camp points.

In the study we compared the size and shape of the cached points and kill/camp points. We did so because the usual ways of determining the function of stone tools—use-wear analysis and residue analysis—were not an option. The reason for this is that most cached points are unused and therefore have neither use-wear nor residues. Our research protocol was further influenced by the fact that the size of cached Clovis points is within the range of variation of the size of historically- and ethnographically-documented hunting weapon points [20]. This is important because it means that size alone cannot be used to determine whether or not cached Clovis points were used for hunting.

In the study we tested two of the predictions of the hunting equipment hypothesis. One concerns point size and shape. If cached points and kill/camp points were produced to arm hunting weapons but the former were mostly unused when they were deposited whereas the latter were mostly used before they were deposited, then cached points should be the same shape as, but generally larger than, kill/camp points. The reason for this is that kill/camp points are likely to be damaged and/or resharpened, and both damage and resharpening inevitably reduce the size of a lithic artifact.

The other prediction involves “allometry,” or size-related shape change. Allometric analysis is commonplace in biology but it has not been used often in archaeology. To date, stone tools have been analyzed allometrically in less than a dozen studies [21]–[30]. The reason for the limited use of allometric analysis in archaeology appears to be that many archaeologists believe that allometry only applies to living things. This is not correct, however. The laws of physics are such that we often have to change the shape of artifacts as we increase or decrease their size. For example, due to the fact that the ratio between the surface area and volume of an object changes as its volume increases, the wings of a large aircraft have to be proportionately larger relative to the fuselage than the wings of a small aircraft. For the same reason, a large sailing ship requires more sail surface area than a small sailing ship to travel at the same rate. In biology, statistically indistinguishable allometric trajectories are taken to be evidence of the same function whereas statistically significant differences in allometric trajectories are taken to be evidence of differences in function [31], [32]. This line of reasoning was also employed in two of the allometry-focused stone tool studies mentioned earlier [23], [26]. Thus, if cached points and kill/camp points were intended for the same purpose, they should follow the same allometric trajectory.

## Materials and Methods

### Sample

Our sample comprised 122 Clovis points. We focused on complete points and specimens missing at most a basal ear because it is difficult to implement the methods we employed with incomplete artifacts. Fifty-four points are from caches. We measured six points from the Anzick cache [11], [33]–[37], 13 from the Drake cache (Colorado; [38]), 14 from the East Wenatchee cache [9], [19], [39], 16 from the Fenn cache (Wyoming; [15]), and five from the Simon cache (Idaho; [8], [40], [41]). It has been suggested that the Anzick points may be burial goods rather than part of a cache, because human skeletal remains have also been recovered at the site [33]–[35]. We do not find this argument convincing for two reasons. First, the artifacts and skeleton were recovered with a front-end loader, so there is no stratigraphic evidence that they are associated [36]. Second, radiocarbon dates derived from some of the artifacts recovered at the site do not overlap with radiocarbon dates derived from some of the human bones, which suggests that they are not contemporaneous [36], [42].

**Figure 1:**
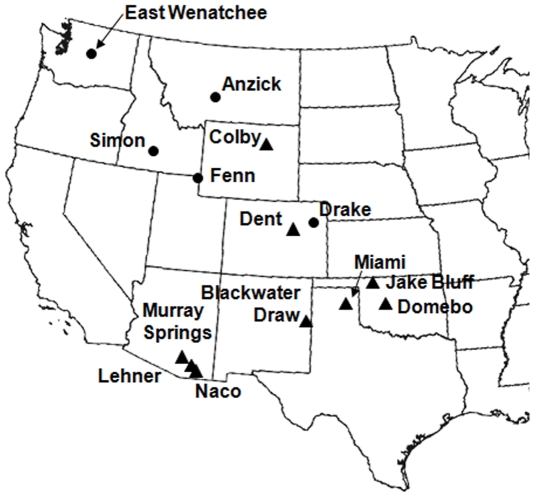
Locations of archaeological sites in the western United States from which points used in the study were recovered. Triangles  =  kill sites/camp sites. Circles  =  caches.

We used epoxy casts in place of five of the points from the Drake cache and one of the points from the East Wenatchee cache. For another three points from the East Wenatchee cache we scanned and digitized technical drawings because neither the actual artifacts nor casts were available for analysis. The locations of the caches are shown in Fig. 1.

The other 68 points in our sample are from kill sites and camps sites. We measured 24 points from Blackwater Draw (New Mexico; [43]–[46]), four from Colby (Wyoming; [47]), two from Dent (Colorado; [48], [49]), four from Domebo (Oklahoma; [50]), and four from Jake Bluff (Oklahoma; [51], [52]). We also measured 10 points from Lehner (Arizona; [53]), three from Miami (Texas; [54], [55]), six from Murray Springs (Arizona; [56], [57]), and eight from Naco (Arizona; [58]). We used epoxy casts in place of three of the points from Blackwater Draw, four from Colby, two from Dent, three from Miami, and two from Naco.

### Digitization

**Figure 2:**
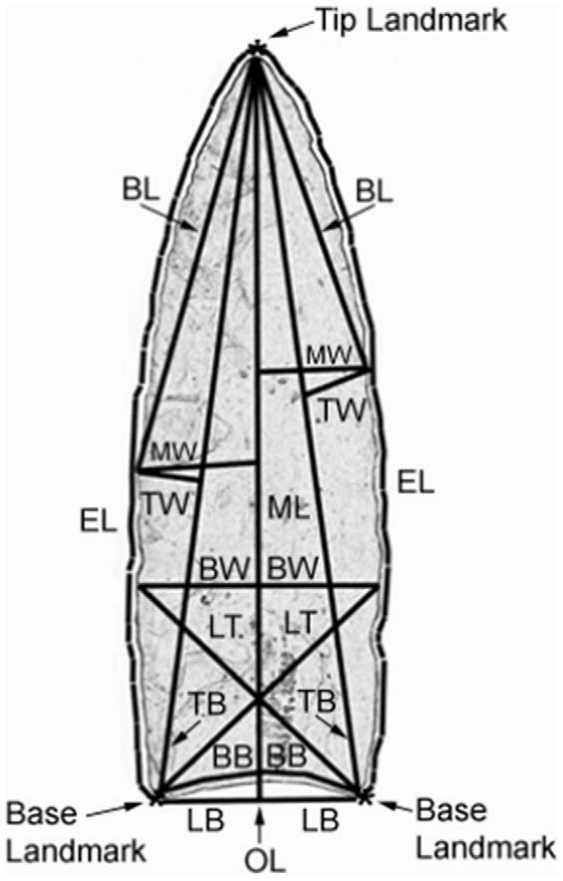
Image of a Clovis point from Blackwater Draw, NM, showing approximate location of characters used in the study. Character abbreviations follow Table 1.

The digitizing method we used was the same as that employed by Buchanan [18], Buchanan and Collard [59], and Buchanan and Hamilton [60]. Digital images of the points were imported into version 2.02 of F.J. Rohlf's Thin Plate Spline Digitizing Program [61] and 32 landmarks placed along the edges and base of each point. The resulting coordinate data were imported into Matlab 6.0 and used to compute 12 characters. The characters are described in Table 1 and illustrated in Fig. 2. Eleven of the characters are interlandmark distances. These were designed to capture the main elements of point form and include traditional linear measurements as well as measurements that cannot be taken easily with calipers but are useful in describing point variation. Four characters relate to the base (BB, LB, BW, and LT), three to the blade length (BL, MW, and TW), and four to overall point length (ML, OL, EL, and TB). The twelfth character is point area (PA). In addition to the 12 characters derived from digitizing the points, thickness was measured directly using calipers or taken from published sources.

**Table 1:** Characters used in the study.

Character	Description
PA	Point area, calculated as the square root of the area enclosed by the 32 landmarks outlining each specimen.
EL	Average of right and left edge-boundary lengths, calculated as the sum of interlandmark distances along the 13 landmarks that define each edge.
TB	Average of the right and left distances from the tip landmark to each of the basal landmarks.
TW	Average of the right and left distances between the tip landmark to basal landmarks (character TB) segments to the position of the maximum edge inflection along each projectile point edge.
BL	Average of the right and left distances between the position of the maximum edge inflection and the tip landmark.
MW	Average of the right and left distances between the positions of the maximum edge inflections to the midline (character ML).
BB	Base boundary length, calculated as the sum of the interlandmark distances along the nine landmarks that define the basal concavity situated between the two basal landmarks.
LB	Base linear length, calculated as the distance between the two basal landmarks.
ML	Midline length, calculated as the distance from the tip landmark to the midpoint of the basal concavity (character BB).
OL	Overall length, calculated as the distance from the tip landmark to the midpoint of the segment between the basal landmarks (character LB).
BW	Basal width at one-third the total length above the basal landmarks.
LT	Average of the right and left distances from basal landmarks to the position at one-third the total length along the opposite edge boundaries.
Thickness	Maximum thickness, taken perpendicular to OL.

The precision of the 12 characters derived from digitizing was estimated on a sample of points from Naco and Lehner. Measurement error—the percentage of the total variance attributable to within-individual variance resulting from imprecision of measurements—was calculated for each character using Model II ANOVA [62]–[64]. Points were chosen randomly and digitized in three non-consecutive sessions, and the variance components were calculated from the resulting dataset. Measurement error associated with the characters ranges from 0.002 to 0.031 percent, which compares favorably to measurement errors reported in biological morphological studies (e.g., [62], [64]). Further, there is no relationship between percent measurement error and the coefficient of variation of a character (*r* = −0.072, *p* = 0.623), which suggests measurement error does not drive variation.

Because the multivariate statistical methods we use require complete data matrices, we estimated missing values for nearly complete points. This was accomplished with the expectation-maximization missing-data replacement method, which uses information about covariation among characters to predict missing values [65]. A simulation-based study has demonstrated that this method is more precise and reliable than principal-component estimation when using a moderate number of characters (6–12) and large sample sizes [65].

As noted, epoxy casts were used in lieu of some of the original points. Buchanan [18] compared epoxy casts of Clovis points from the Lehner site to the actual points and found that there was no statistical difference between the casts and the real artifacts. The paired *t*-tests he carried out gave *p* values ranging between 0.841 and 0.962. Consequently, the inclusion of epoxy casts in the sample is not expected to have affected the study.

### Analyses

To test the prediction that cached points should be the same shape as, but generally larger than, kill/camp points, we used histograms and principal components analysis (PCA). The histogram analysis focused on the second part of the prediction, namely that cached points should be generally larger than kill/camp points. To test this part of the prediction, we plotted point area (PA) and overall length (OL) separately for the kill/camp points and the cached points, and then compared the histograms on the same scale.

The PCA was designed to test both parts of the prediction. We opted for PCA because it is generally accepted to be capable of decomposing form into size and shape by researchers who work with morphometric data (e.g., [66]). In studies in which PCA is applied to morphometric data, it is usual for the first principal component (PC1) to be assumed to reflect size variation among the taxa and for the other components, which are orthogonal to and therefore uncorrelated with PC1, to be taken to reflect shape variation among the taxa.

We began by log-transforming the data to make them more closely approximate a normal distribution [67]. We then tested the 13 characters for normality with the Kolmogorov-Smirnov test. Having found that the characters did not depart significantly from normality, we subjected them to PCA. We retained the principal components that constituted more than 1% of the variation in the dataset. We then used the *t*-test to evaluate the significance of the differences between the cached points' and kill/camp points' scores on the retained principal components. In line with the standard interpretation of morphometrics-derived PCs discussed above, the test prediction was that the scores for the cached points and kill/camp points should differ significantly on PC1 but not on any other PC.

To test the prediction that cached points and kill/camp points should follow the same allometric trajectory, we conducted bivariate analyses of allometry using point area as a proxy for point size. We used point area instead of the more traditional measure of point size—overall length—because the latter character is more susceptible to disproportionate reduction as a result of resharpening [22]. We used a simple model of allometric shape change, *y* = *bX^k^*, where *X* and *Y* are the sizes of two forms and *k* (the allometric coefficient) is an exponential factor that relates the sizes. The allometric equation *y* = *bX^k^* is commonly used in the logarithmically transformed form that translates to a simple linear relationship, log*_e_Y* = log*_e_b*+*k* log*_e_X*, where *Y* is the character examined in relation to size, *k* is the slope, or allometric coefficient, and *b* is the y-intercept [68]. Using a linear-regression model, the regression coefficient of logged *Y* on logged *X* is an estimate of *k*, the allometric coefficient [69]. We used least squares regression because it is the most conservative of the estimation procedures that have been advocated for bivariate allometry analysis [70].

Allometric coefficients (*k*) indicate the manner in which given characters change in relation to point size. Values greater than unity (*k*>1) indicate positive allometry (characters disproportionately larger relative to size), and values less than unity (*k*<1) indicate negative allometry (characters disproportionately smaller relative to size). Isometry is a property of characters that increase at the same relative rate with proportions remaining constant (*k* = 1) [71]. For the allometric analyses, we tested for homogeneity in the allometric coefficients (*k*) and the y-intercepts (*b*) associated with cached points and kill/camp points. We used Benjamini and Yekutieli's [72] method of significance-level correction for multiple comparison tests. Narum [73] has shown that Benjamini and Yekutieli's [72] method optimizes the reduction of both Type-I and Type-II error rates.

We conducted the histogram analysis, Kolmogorov-Smirnov tests, and the *t*-tests in PASW (SPSS) 18. All other analyses were conducted in MATLAB 6.0 (release 12), using statistical functions written by R. E. Strauss [74].

## Results

### Test of the prediction that cached points should be the same shape as, but generally larger than, kill/camp points

**Figure 3:**
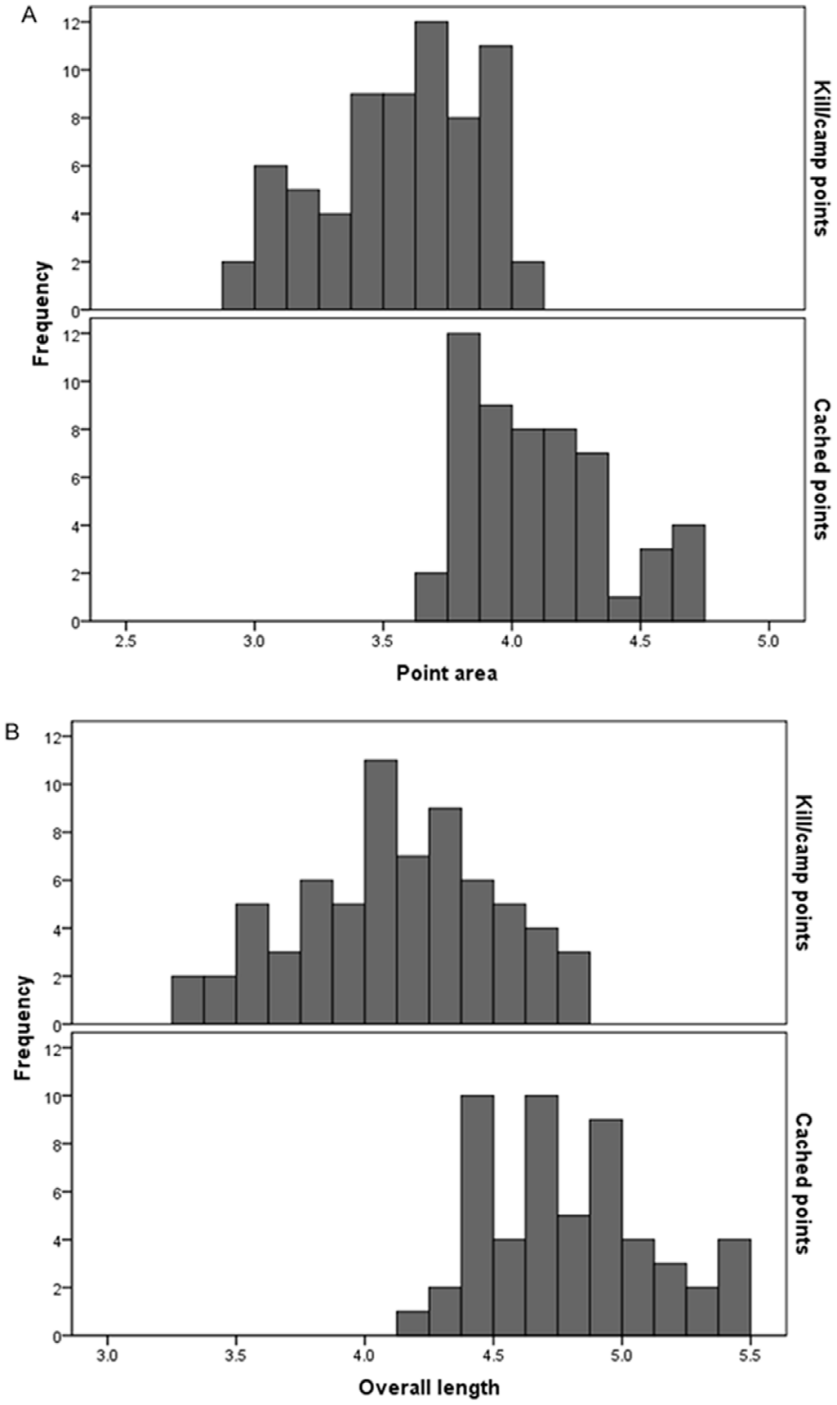
Histograms comparing size characters between kill/camp points and cached points. A) point area. B) overall length.

The histograms generated to test the size part of the prediction are shown in Figure 3. The histograms are consistent with the prediction. For both characters, there is some overlap between the cached points and the kill/camp points but all the large points are from caches and all the small points are from kill/camp sites.

**Figure 4:**
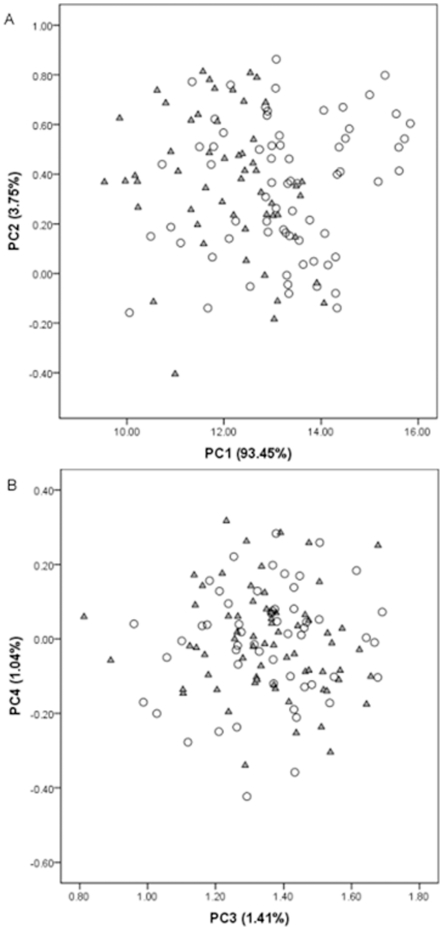
Bivariate plots of principal component scores. A) PC1 versus PC2. B) PC3 versus PC4. Triangles  =  kill/camp points. Circles  =  cached points.

The results of the PCA conducted to test the prediction that cached points should be the same shape as, but generally larger than, the kill/camp points are summarized in Figure 4 and Table 2. Four principal components met the criterion for retention. As expected, PC1 accounts for the majority of variation in the dataset (93.5%). The large magnitude and consistent direction of the loadings of the 13 characters on PC1 is consistent with the idea that it represents size variation (Fig. 4a). The other three principal components capture aspects of shape variation, as expected. The loadings on PC2 (3.75% of the variation) indicate a changing relationship between width (TW, MW, and BW) and base characters (BB and LB) on the one hand and length characters (EL, TB, ML, and OL) on the other, such that in Fig. 4a points become shorter and wider as one moves up the PC2 axis. The loadings on PC3 (1.41% of the variation) indicate a changing relationship between base characters (BB and LB) and width characters (TW and MW). As shown in Fig. 4b, points become more triangular (wider bases, narrow tips) as one moves from left to right along the PC3 axis. Lastly, the loadings on PC4 (1.04% of the variation) contrast BL with TW such that points become narrower with long blades as one moves up the PC4 axis (Fig. 4b).

**Table 2:** Results of principal components analysis of combined sample of points.

Character	PC1	PC2	PC3	PC4
*% Variation*	93.45	3.75	1.41	1.04
PA	0.997	0.027	−0.013	−0.056
EL	0.990	−0.126	0.001	−0.038
TB	0.988	−0.147	0.012	−0.037
TW	0.897	0.337	−0.247	−0.126
BL	0.954	−0.081	−0.073	0.277
MW	0.916	0.361	−0.137	0.087
BB	0.902	0.290	0.291	0.028
LB	0.902	0.278	0.308	0.018
ML	0.985	−0.162	−0.001	−0.046
OL	0.987	−0.154	−0.005	−0.044
BW	0.946	0.307	−0.018	0.013
LT	0.997	0.017	0.055	−0.021
Thickness	0.995	0.015	0.058	−0.027

The distribution of points in Fig. 4a suggests that cached points tend to be larger than kill/camp points. In this figure only cached points have PC scores above 14 on the PC1 axis, which represents size, whereas both cached and kill/camp points overlap below 14 on the PC1 axis. This is supported by the results of the *t*-test of the PC1 scores for the two groups of points. According to this *t*-test, the difference between the PC1 scores for the cached points and the kill/camp points is highly significant (*t* = −10.82, *df* = 120, *p*<0.000). In contrast, there is no difference between cached points and kill/camp points on the other PCs. Along the PC2 axis, cached points and kill/camp points exhibit considerable overlap, and the *t*-test confirms that there is no statistical difference in PC2 scores between the two groups of points (*t* = 0.79, *df* = 120, *p* = 0.429). Overlap of cached points and kill/camp points is also evident in the plot of the PC3 and PC4 scores (Fig. 4b). In line with this, scores from PC3 and PC4 are not different between the two groups of points, according to the *t*-test (*t* = −0.01, *df* = 120, *p* = 0.989 and *t* = 0.03, *df* = 120, *p* = 0.977, respectively). Thus, the results of the PCA are consistent with the prediction that cached points should be the same shape as, but generally larger than, kill/camp points.

### Test of the prediction that cached points and kill/camp points should follow the same allometric trajectory

**Figure 5:**
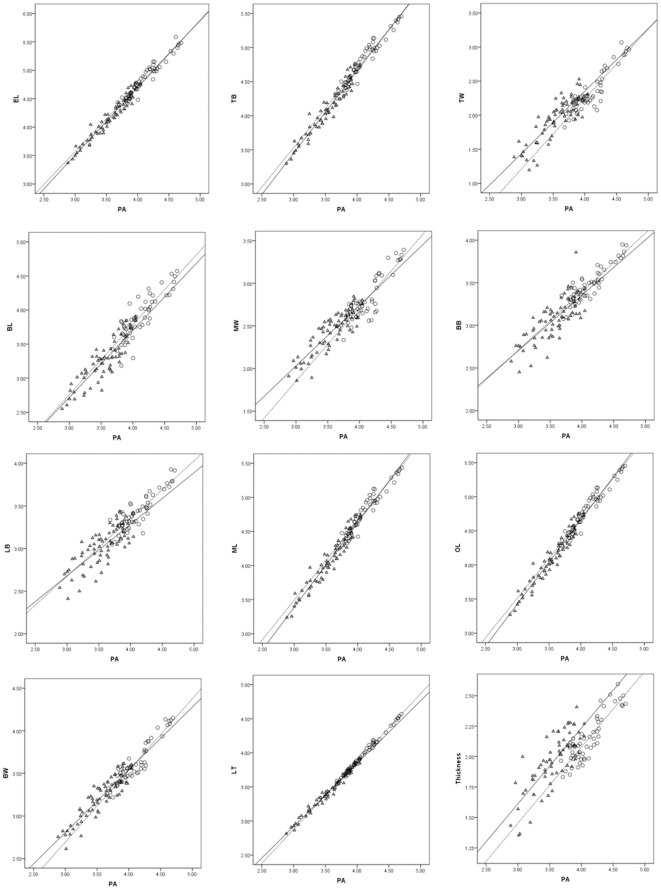
Bivariate plots of characters against point area. Character abbreviations follow Table 1. Triangles  =  kill/camp points. Circles  =  cached points. Solid line  =  best-fit line for kill/camp points. Dashed line  =  best-fit line for cached points.

Table 3 and Figure 5 summarize the results of the analyses carried out to test the prediction that cached points and kill/camp points should follow the same allometric trajectory. Points from caches have positive allometric coefficients for the characters EL, TB, TW, BL, ML, and OL and have negative allometric coefficients for the characters MW, BB, LB, BW, and thickness. Thus, all the length characters (EL, TB, BL, ML, and OL) and one width character (TW) tend to be larger relative to point area in large points than in small points. In contrast, another width character (MW), the three basal characters (BB, LB, and BW), and thickness tend to be smaller relative to point area in large points than in small points. The third width character (LT) is very close to isometry in the cached points, indicating that it increases more or less proportionally with overall size.

**Table 3:** Results of analyses in which characters were regressed on point area to test the prediction that cached points and kill/camp points should follow the same allometric trajectory.

	Cached points	Kill/camp points		Cached points	Kill/camp points	
Character	y-intercept	y-intercept	p-value^a^	*k* b	*k* b	p-value^a^
EL	0.112	−0.057	0.416	1.152	1.187	0.512
TB	0.071	−0.168	0.280	1.154	1.206	0.371
TW	−1.869	−1.317	0.117	1.027	0.918	0.241
BL	−0.314	−0.226	0.826	1.025	0.982	0.682
MW	−0.737	−0.105	0.037	0.862	0.712	0.056
BB	0.653	0.759	0.737	0.686	0.649	0.666
LB	0.645	0.864	0.480	0.677	0.605	0.387
ML	0.014	−0.335	0.135	1.160	1.241	0.188
OL	0.026	−0.273	0.181	1.163	1.233	0.236
BW	0.174	0.601	0.067	0.840	0.735	0.087
LT	−0.105	0.154	0.002*	0.995	0.922	0.002*
Thickness	−0.423	−0.251	0.560	0.625	0.621	0.959

a p-values are from ANOVA tests for heterogeneity of y-intercepts and slopes between cached points and kill/camp points.

b *k* = allometric coefficient;

* significant difference between coefficients using Benjamini and Yekutieli's (2001) alpha correction (the critical value for 24 tests is α = 0.0132).

Kill/camp points have positive allometric coefficients for length characters (EL, TB, ML, and OL), indicating that these characters tend to be longer relative to point area in large points than in smaller points. Conversely, kill/camp points have negative allometric coefficients for the characters TW, BL, MW, BB, LB, BW, and thickness. Therefore, width (MW and TW), blade length (BL), basal characters (BB, LB, and BW), and thickness tend to be smaller relative to point area in larger points than in smaller points. The character LT also is very close to isometry in the cached points. Again, this indicates that LT basically increases proportionally with overall size.

Visual inspection of the allometry plots suggests that there are no allometric trajectory differences between the cached points and kill/camp points (Fig. 5). Results of the statistical comparisons of the y-intercepts and slopes for cached points and kill/camp points are consistent with this for 11 of the 12 characters (Table 3). For the remaining character, LT, the slope for cached points is significantly closer to 1 than the slope for kill/camp points, and the y-intercept for cached points is significantly lower than the y-intercept for kill/camp points (Table 3). Given that cached points and kill/camp points follow the same allometric relationship in all but one of the characters examined, we consider the results of the allometric analyses to be consistent with the prediction that cached Clovis points and Clovis points from kill and camp sites should follow the same allometric trajectory.

## Discussion

### Do the results support the hunting equipment hypothesis?

We compared morphometric data from cached Clovis points and Clovis points from kill and camp sites to test the following predictions of the hunting equipment hypothesis: 1) cached points should be the same shape as, but generally larger than, kill/camp points, and 2) cached points should follow the same allometric trajectories as kill/camp points. The results of the analysis carried out to test the first prediction were entirely consistent with it. The results of the analysis carried out to test the second prediction showed that cached points and kill/camp points follow the same allometric relationship in all but one of the characters examined. Together, these results support the idea that cached Clovis points were intended to arm hunting weapons.

### Do the results support any of the other hypotheses?

Earlier we outlined a number of other hypotheses that have been offered to explain the function of cached Clovis points. To reiterate, one of these contends that points from a number of Clovis caches were produced for use in rituals rather than for hunting [12]–[17]. The other two hypotheses were put forward to account for the large points from the East Wenatchee cache. One holds that the points in question were the focus of costly signaling displays [1], [18]. The other contends that they were too big to have been used for hunting and were intended to be used as saws instead [19].

Although we did not directly test these other hypotheses, our results have a bearing on them. To begin with, our results suggest that the costly signaling hypothesis can be discounted as an explanation not only for cached Clovis points in general but also for the points that gave rise to the hypothesis in the first place—the points from the East Wenatchee cache. The general version of the costly signaling hypothesis predicts that there should be differences in the allometric trajectories of the characters between cached Clovis points and Clovis points from kill and camp sites. The reason for this is that the hypothesis holds that Clovis point makers who engaged in costly signaling would have attempted to produce points with exaggerated length and width dimensions because it is difficult to create long and wide points without increasing thickness. This in turn would lead length and width characters to have a different relationship to point area in cached points versus kill/camp points without any difference between the two groups of points in the relationship between thickness and point area. Crucially with respect to this hypothesis, our analyses indicate that the allometric trajectories of blade length, maximum width, and thickness do not differ between the cached Clovis points and the kill/camp Clovis points. Thus, our analyses suggest that the costly signaling hypothesis can be discounted as a general explanation for the function of cached Clovis points, at least in the form proposed by Buchanan [18] and Kilby [1].

If the costly signaling hypothesis is restricted to points from the East Wenatchee cache, the prediction is that there should be differences in the allometric trajectories of the blade length characters between the points from the East Wenatchee cache and all the other Clovis points in the sample. Our analyses are not consistent with this prediction either. The East Wenatchee points do not differ significantly from the other cached Clovis points or the Clovis kill/camp points in terms of the allometric coefficients of the characters examined (Table 4). The y-intercepts for the characters EL and LT are different between the East Wenatchee cache and all the other Clovis points in the sample, but the difference is the reverse of what the costly signaling hypothesis predicts. Specifically, the y-intercept for the East Wenatchee cache is lower, not higher, than the y-intercept for the other Clovis points. Thus, our analyses also suggest that in its current form the costly signaling hypothesis can be discounted as an explanation for the function of the points recovered from the East Wenatchee cache.

**Table 4:** Results of analyses in which characters were regressed on point area to compare the allometric trajectories of the points from East Wenatchee and the other points in the sample.

	East Wenatchee points	Other points		East Wenatchee points	Other points	
Character	y-intercept	y-intercept	p-value^a^	*k* b	*k* b	p-value^a^
EL	−1.063	−0.249	0.004*	1.404	1.244	0.027
TB	−0.834	−0.395	0.125	1.344	1.272	0.342
TW	−1.385	−0.790	0.223	0.935	0.762	0.177
BL	−1.489	−0.465	0.101	1.288	1.055	0.136
MW	−0.164	0.185	0.334	0.754	0.628	0.186
BB	1.043	0.709	0.453	0.606	0.665	0.612
LB	1.118	0.756	0.397	0.580	0.639	0.601
ML	−0.873	−0.535	0.262	1.346	1.300	0.558
OL	−0.823	−0.462	0.205	1.340	1.288	0.486
BW	0.703	0.753	0.865	0.735	0.692	0.570
LT	−0.326	−0.001	0.008*	1.041	0.967	0.024
Thickness	0.672	0.350	0.468	0.391	0.442	0.662

a p-values are from ANOVA tests for heterogeneity of y-intercepts and slopes between points from East Wenatchee and points from the other assemblages.

b *k* = allometric coefficient;

* significant difference between coefficients using Benjamini and Yekutieli's (2001) alpha correction (the critical value for 24 tests is α = 0.0132).

We think the results of our analyses also shed light on the saws-not-hunting-weapons hypothesis. As with the costly signaling hypothesis, it is possible to treat the saws-not-hunting-weapons hypothesis as a general explanation for cached Clovis points and as a specific explanation for the points from the East Wenatchee cache. And as with the costly signaling hypothesis, we believe our results allow both versions of the hypothesis to be discounted. Given that the allometric trajectories of the majority of the characters we examined do not differ between the cached Clovis points and kill/camp Clovis points, there is no reason to believe that the function of the two groups of points differed. When this is taken together with the fact that some of the kill/camp points were clearly used for hunting, it suggests that the cached Clovis points were not intended to be saws. It is more parsimonious to assume that they were intended to be the tips of hunting weapons.

The same logic holds for the East Wenatchee points. As noted in the previous paragraph, they do not differ from the other points in the sample with respect to the allometric trajectories of the characters examined. Hence, in view of the fact that we know that some of the other points were used for hunting, there is no reason to believe that they were intended to be used as saws. Of course, we cannot rule out the possibility that the East Wenatchee points might have been used as saws, but our results suggest that any such use would have been incidental to their intended function.

Three hypotheses with a ritual element can be identified in the literature on Clovis caches [1], [11]–[17]. However, two of these hypotheses are not relevant for present purposes. The two hypotheses in question were put forward by Wilke et al. [11] and Kilby [1], respectively. Wilke et al. [11] speculated that caches that are associated with burials include tools that were intended to be functional and were included in the burial to help the deceased reproduce the technological system in the afterlife. Kilby [1] argued that some caches consist of items that were being used by the interred individual and/or other members of their group. The reason these hypotheses are not relevant for present purposes is that they both assume that cached points were intended to be parts of hunting weapons and that it was only the deposition of the points that was ritual. Thus, they do not represent real alternatives to the hunting equipment hypothesis.

The third hypothesis with a ritual element was first proposed by Frison and Bradley in the late 1990 s [15]. Since then it has been offered as an explanation for certain cached points by Bradley and Stanford [13], Gillespie [16], Bamforth [12], Ellis [14] and Speth et al. [17]. According to this hypothesis, the large points in some Clovis caches were produced specifically for ritual purposes. Critically, then, this hypothesis views ritual as the intended goal of production rather than as something that is incidental to the production of the points, as is the case with Wilkes et al.'s [11] and Kilby's [1] hypotheses. This makes it a direct competitor to the hunting equipment hypothesis when it comes to explaining the form of cached Clovis points.

Do our results support this hypothesis? We think not. Again, given that for the vast majority of the characters we examined there is no significant difference between the allometric trajectories of the cached points and the kill/camp points, there is no reason to invoke a different intended purpose for the cached points compared to the kill/camp points. Since the kill/camp points are known, or can reasonably be inferred, to have been used for hunting, the simplest hypothesis is that both groups of points were intended to be used as parts of hunting weapons.

In sum, then, the results of our analyses do not support any of the other hypotheses that have been put forward to account for the function of cached Clovis points. They only support the hunting equipment hypothesis.

### On the principles that guided the production and maintenance of Clovis points

If we assume for the sake of argument that the results of our study are conclusive and that the primary function of Clovis points was to arm hunting weapons, the data presented here can be used to shed some light on the principles that guided the production and maintenance of Clovis points. Given that many of the cached points appear to be unused whereas many of the kill/camp points clearly have been used, allometric analyses of the combined sample can provide a use-life trajectory for Clovis points.

When allometric analyses are run with the two groups of points combined, 11 of the 12 characters return slopes that are significantly different from 1 when they were regressed on point area (Table 5). The length characters (EL, TB, ML, and OL) are positively allometric, whereas the width and basal characters (TW, MW, BB, LB, LT, and BW) are negatively allometric. Thickness is also negatively allometric. The only character that returns a slope that does not differ from 1.0 is BL (*F* = 1.45, *df* = 1,107, *p* = 0.239).

**Table 5:** Results of analyses in which characters from the combined sample of points were regressed on point area to assess the characters' allometric trajectories.

Character	y-intercept	r^2^	*k* a	Std. Error	p-value^b^
EL	−0.093	0.970	1.200	0.019	<0.000*
TB	−0.198	0.965	1.217	0.021	<0.000*
TW	−1.101	0.831	0.849	0.035	<0.000*
BL	−0.512	0.873	1.067	0.037	0.041
MW	−0.182	0.849	0.731	0.028	<0.000*
BB	0.599	0.809	0.696	0.031	<0.000*
LB	0.593	0.813	0.685	0.030	<0.000*
ML	−0.320	0.963	1.239	0.022	<0.000*
OL	−0.252	0.966	1.229	0.021	<0.000*
BW	0.507	0.909	0.760	0.022	<0.000*
LT	−0.012	0.991	0.970	0.009	<0.000*
Thickness	0.197	0.670	0.485	0.031	<0.000*

a *k* = allometric coefficient.

b p-values indicate if slopes are significantly different from 1.

* significantly different from a slope of 1 using Benjamini and Yekutieli's (2001) alpha correction (the critical value for 12 tests is α = 0.0161).

We suspect that the positive allometry of characters EL, TB, ML, and OL is driven primarily by resharpening, which during the early Paleoindian period appears to have focused on the tips of points that were damaged or dulled through use. The shape of Clovis points is such that removing flakes from the tip of a point would have altered the length of the point more dramatically than it altered the area of the point. Hence, in a sample comprising unused and retouched Clovis points, characters that reflect length should decrease more rapidly than point area. The corollary of this is that the relationship between the length characters and point area should be positively allometric.

We can think of two factors that may have—singly or collectively—produced the negative allometry of the width and basal characters, and of thickness. The first is resharpening. Because resharpening focused on point tips, it will have reduced the length of a point as well as its area, but may not have decreased the width of the point, the dimensions of its base, or its maximum thickness. One consequence of this is that when width and basal characters and thickness are subjected to allometric analyses in which point area is used as the proxy for size, the resulting relationships are likely to be negatively allometric. The other factor that may have produced negative allometry in the width, basal, and thickness characters is the functional constraint of hafting. It seems likely that the width and thickness of larger points would have been kept relatively narrow and thin to facilitate hafting to a foreshaft. This too can be expected to result in negative allometry when width and basal characters and thickness are subjected to allometric analyses in which point area is used as the proxy for size, because larger points will have relatively smaller widths and thicknesses than smaller points.

The character that returns a slope that does not differ from 1.0, BL, measures the leading edge of the point. This is the section of the point that penetrates the hide and flesh of prey. The isometry of BL (Fig. 5) suggests that for any given size of point, Clovis hunters aimed to maintain a particular proportion of blade length. This was true even with the smallest of Clovis points in the sample, suggesting that even after point tips were resharpened or reworked, blade length was kept proportional. Interestingly, Buchanan [22] found similar allometric trajectories in a sample of Folsom points from the Southern Plains. We suspect that this property may have been optimal for the tip's penetration into the hide and flesh of prey. It also might have minimized the likelihood of catastrophic breakage when a point was in use.

Light is potentially also shed on the construction and maintenance of Clovis hunting weapons by the fact that character LT had a different allometric trajectory in cached points than in kill/camp points. To reiterate, both groups of points returned negative slopes for LT, but the slope returned by the kill/camp points was more negatively allometric than the slope for the cached points. Given that LT runs from the basal ears to one-third up the edge means that the lower third of the edges of cached points is slightly but consistently more convex than the equivalent part of kill/camp points.

The allometric coefficients for LT in the cached point and kill/camp point samples are both very close to 1, and the difference between the slopes for the two groups of points cannot be seen in the relevant scatter plot (Fig. 5). As such, we do not want to make too much of this finding. However, there is one possibility that is worth considering. Given that the allometric trajectories of the basal, thickness, and non-LT width characters do not differ between the cache points and kill/camp points, the difference between the cached points and kill/camp points in the allometric coefficients for LT must be due to differences in the shape of the distal two-thirds of the edges of the points. More specifically, it must be due to the kill/camp points having straighter or more concave edges between LT's distal landmark and the tip than the cached points. One way in which this could have come about is if Clovis points were hafted in such a way that about a third of their length was embedded in the spear shaft and covered with binding, and used points were resharpened while they were hafted. Thus, the difference between the cached points and kill/camp points in the allometric coefficients for LT may tell us something important about the construction of Clovis hunting spears and about how such weapons were maintained.

### Possibilities for future research

With regard to further research, there are two obvious next steps. The first is to use experiments to shed light on the design aspects of Clovis points discussed in the previous section. One set of experiments could focus on the resharpening hypothesis. Using the combined sample of Clovis points, we found that length characters exhibited positive allometry, whereas the width and basal characters, and maximum thickness showed negative allometry. We proposed that these allometric relationships were produced primarily through resharpening. This hypothesis can be tested by repeatedly resharpening replica Clovis points, measuring them after each resharpening event, and then carrying out allometric analyses in which the length, width, base, and thickness characters are regressed on point area. If the hypothesis is correct, then the point length characters should exhibit positive allometry and the width, base, and thickness characters should display negative allometry. Shott et al. [30] conducted a similar type of experimental analysis using replica Folsom points.

Another set of experiments could focus on blade length. Our results indicate that Clovis point blade length displays an isometric trajectory, and we suggested that this was the result of blade length needing to be a certain proportion of point size for optimal functioning. An experiment using a set of replica Clovis points of varying sizes and blade lengths could be used to test the performance characteristics associated with blade lengths with negative, positive, and isometric allometric trajectories. Point performance could be assessed by firing them into carcasses following the procedures of Frison [75] and Cheshier and Kelly [76] and then determining which set of points experience the least amount of breakage. Point performance could also be assessed using Waguespack et al.'s [77] experimental launching procedure using the replica points. Their experiment used a remotely triggered compound bow from a specified distance to assess the accuracy, precision, and depth of penetration of different points being fired at a target. Our hypothesis predicts that replica points with isometric blade lengths should outperform points with blade lengths that have negative or positive allometric trajectories.

Given that our results suggest cached points were intended to be used as parts of hunting weapons, the other obvious next step is to determine how caches fitted into the food procurement and land use strategies of Clovis groups. Currently, there are two competing models. The first, suggested by Collins [78], is that caching of hunting equipment was the result of the predictable use of the landscape. In this model, Clovis hunters deposited caches of tools because they expected to return to particular spots on the landscape and the caching of hunting equipment would have allowed them to replenish their toolkits without having to return to a raw material source location. The other model was proposed by Meltzer [10]. He suggested that caches were used by Clovis groups to help them explore new territories by establishing supply depots between known and unknown landscapes. In this model, Clovis hunters exploring a new landscape would have cached hunting equipment as they moved away from known sources. This behavior would have reduced the risk of moving into unknown landscapes. One way of testing between these models is to obtain radiocarbon dates from more caches, and then compare the cache dates to the earliest dates from non-cache Clovis sites. Meltzer's aides-to-exploration hypothesis clearly predicts that the caches should not only be indistinguishable from the earliest non-cache Clovis sites in terms of their temporal distribution, but also exhibit a similar geographic pattern to the earliest non-cache Clovis sites, which Hamilton and Buchanan [79] have shown are consistent with a population diffusion from the Ice-Free Corridor. In contrast, Collins' [78] hypothesis predicts that caches will occur throughout the entire Clovis period and will not be significantly correlated with distance from any of the points of entry into North America that have been proposed for the Clovis Paleoindians.

### Summary

In the study reported here we tested two predictions of the hypothesis that cached Clovis points were intended to be used as parts of hunting weapons. The two predictions are: 1) cached points should be the same shape as, but generally larger than, points from kill/camp sites, and 2) cached points and kill/camp points should follow the same allometric trajectory. The results of the analyses are consistent with both predictions and therefore support the hunting equipment hypothesis. Significantly, the results of the analyses are not consistent with the predictions of the other hypotheses. Thus, we contend that cached Clovis points were likely produced with a view to them being used to arm hunting weapons.
